# The Effects of Aflatoxin B1 Intake in Assaf Dairy Ewes on Aflatoxin M1 Excretion, Milk Yield, Haematology and Biochemical Profile

**DOI:** 10.3390/ani13030436

**Published:** 2023-01-27

**Authors:** Raúl Bodas, F. Javier Giráldez, Sara Olmedo, Marta Herrera, Susana Lorán, Agustín Ariño, Secundino López, Alberto Benito, Teresa Juan

**Affiliations:** 1 Agrarian Technological Institute of Castile and Leon (ITACyL), Ctra. Burgos, Km 119, 47071 Valladolid, Spain; 2 Instituto de Ganadería de Montaña (CSIC-Universidad De León), Finca Marzanas s/n, 24346 Grulleros, Spain; 3 Facultad de Veterinaria, Instituto Agroalimentario de Aragón-IA2 (Universidad de Zaragoza-CITA), 50013 Zaragoza, Spain; 4 Centro de Investigación y Tecnología Agroalimentaria de Aragón (CITA), Avda. Montañana 930, 50059 Zaragoza, Spain

**Keywords:** aflatoxin B1, aflatoxin M1, milk, dairy ewes, Assaf, carryover, haematology, blood biochemical parameters

## Abstract

**Simple Summary:**

Aflatoxin B1 is a mycotoxin produced by toxigenic moulds that contaminate feedstuffs. If aflatoxin B1 is ingested by ewes, they can get sick and aflatoxin M1 can be found in milk. The objective of this work was to study the transfer of different moderate doses of aflatoxin B1 ingested by Assaf ewes (40 or 80 μg aflatoxin B1/day) into milk (aflatoxin M1) and its effect on animals’ health and performance. There is a clear transfer of aflatoxin B1 (feed) into aflatoxin M1 (milk). The transfer rate depends on the aflatoxin B1 dose (the lower the dose, the higher the transfer rate) and milk yield (high-producing animals show higher transfer rates than low-producing ones). Ewes’ milk yield and health were not visibly affected.

**Abstract:**

The aim of this study was to investigate the in vivo transfer of aflatoxin B1 (AFB1) to Assaf ewes’ milk (aflatoxin M1, AFM1) and its effect on animal performance and health. Thirty Assaf ewes were allocated to three groups (C, L, H), and received a different individual daily dose of AFB1 (0, 40 and 80 μg) for 13 days. Milk (days 1, 2, 3, 4, 7, 14, 16 and 18) and blood (days 1, 7, 14 and 18) samples were collected. Milk yield, composition (except protein) and somatic cell counts (SCC) were not affected by AFB1 intake (*p* > 0.05). Haemoglobin concentration increased (*p* < 0.05) and haematocrit and alanine aminotransferase levels tended to increase (*p* < 0.10) in group H on day 14. AFM1 excretion was highly variable and detected in L and H animals from days 1 to 16 (3 days increase, 10 days steady-state, 3 days clearance). Carry-over rate (0.23%) was significantly higher in L (0.22–0.34%) than in H (0.16–0.19%) animals (*p* < 0.05). AFB1 daily doses of 40 to 80 µg do not impair milk yield; however, it may start affecting animals’ health. Milk AFM1 depends mainly on the AFB1 intake whereas carryover rate is positively influenced by the level of milk production.

## 1. Introduction

Aflatoxins are mycotoxins produced by toxigenic strains of *Aspergillus flavus* and *A. parasiticus* that may contaminate foodstuffs. Aflatoxin M1 (AFM1) is the hydroxylated metabolite of aflatoxin B1 (AFB1) and can be found in milk and derived products obtained from livestock that have ingested contaminated feed. The contamination of food and feed with mycotoxins is a global threat to food safety and has great public health and economic significance. The European Food Safety Authority (EFSA) is continuously updating its risk assessment to advise the EU Commission about the need for new limits and/or modifications to the existing maximum contents in food and feed.

The presence of AFM1 in milk in the EU should not be considered a major public health concern as there are strict regulations in place to protect the health of consumers. Furthermore, several screenings carried out on milk and dairy products [1,2,3,4] have shown that levels are controlled. Thus, although AFB1, which is the precursor of AFM1, is present in up to 30% of cattle feed, the amounts are below the maximum limit established in the legislation, with very few positive cases [2,4,5,6].

However, outside the EU, the number of AFB1-positive feed samples can be much higher [7]. In this regard, the import of feedstuffs from third countries in a food shortage scenario may increase the frequency of AFB1-positives. In this regard, the impact of the Russia–Ukraine conflict on agri-food products trade has been immediate, inducing critical shortages of animal feed that may be addressed by securing imports from other origins [8]. 

Aflatoxins possess a high acute and chronic toxicity, including genotoxic and carcinogenic effects on animal and human health [9]. Ruminants have a higher resistance to aflatoxicosis than other animals; however, their ability to inactivate AFB1 is severely limited [9], and this mycotoxin can affect the animals’ biochemical profile. For example, increases in aspartame transaminase (AST), alanine transaminase (ALT), alkaline phosphatase (ALP), malondialdehyde (MDA) and lipid peroxidation as well as decreases in gamma-glutamyl transferase (GGT) have been reported in response to mycotoxin intake [10,11].

The main target organ of aflatoxins in animals is the liver, whose adverse effects have been reported in the form of acute intoxication characterized by severe liver damage, anorexia, jaundice, weight loss, gastrointestinal disorders, haemorrhage, oedema and even death. Chronic sublethal exposure leads to immunosuppression, nutritional dysfunctions and cancer. Several studies on AFM1 occurring in milk reported carcinogenic and immunosuppressive effects similar to those of AFB1, both in humans and in other animals, although with a less potent effect [12]. AFM1 is the only mycotoxin for which maximum levels in milk have been established. Lactocytes also have some ability to transform AFB1 into AFM1, which transports both by passive diffusion and via an active xenobiotic transporter into the lumen of the mammary alveolus, allowing high concentrations to be reached in short periods of time [9]. Moreover, a recent study has shown that, depending on the concentration of spores and fungi present in the feed, aflatoxin production may be also possible in the rumen of the animals [13].

The transfer rate of AFB1 from feeds to AFM1 in milk is highly variable, with values ranging from 0.6 to 6% in cows [6]. The transfer rate in sheep is generally lower, with values ranging from 0.08 to 0.33% in Sarda ewes [14,15] to 0.54% in Lacaune ewes [16]. Nevertheless, the transfer rate is influenced by various pathophysiological factors, including feeding regime, health status, individual biotransformation capacity and volume of milk production. In this regard, the expression of the BCRP/ABCG2 transporter, present in the luminal part of the lactocytes and contributing to the excretion of AFM1 in milk, seems to increase with the production potential of the animal [9].

Sheep milk production is typical in the Mediterranean region, and Spain is one of the main producers with an approximate production of 536,000 t/yr [17]. Almost 45% of the Spanish dairy industry is located in in Castile and Leon (northwest Spain), which produces 54% of the total ewe milk production of Spain [17,18]. Spanish Assaf is currently the most important dairy sheep breed in Spain. This breed has undergone a process of adaptation and selection over the last 35 years to achieve sustained high levels of production over time [19]. These animals are heavier and with higher milk production potentials than the Sarda [14,15] and Lacaune ewes [16]. However, there are no available data in the literature concerning the rate of transfer of AFB1 to AFM1 or the average excretion of AFM1 in milk in Assaf ewes.

Therefore, the aim of the present work was to study the in vivo transfer of AFB1 ingested by Assaf ewes into milk (detected as AFM1) and its effect on the productive performance and biochemical profile and haematic parameters.

## 2. Material and Methods

### 2.1. Animal Ethics

The experiment was compliant with the Directive 2010/63/EU of the European Parliament and of the Council of 22 September 2010 on the protection of animals used for scientific purposes. The experimental procedures were approved by the Institutional Animal Care and Use Committee of the Agrarian Technological Institute of Castilla y León (ITACyL, Spain) and the competent authority (Directive 2010/63/EU) under the protocol number 2017/25/OH.

### 2.2. Animals and Diets

Thirty lactating Assaf ewes in week 4 of lactation were used (average body weight ± standard deviation = 80.0 ± 8.23 kg). Animals were allocated to three experimental groups balanced by body weight and milk yield at the experimental farm of the Instituto de Ganadería de Montaña (CSIC-Universidad de León) located in the northwest of Spain (Castilla y León). The ewes were individually housed, fed and milked, and were able to hear and see other sheep. All the animals received during the whole experimental period the same total mixed ration (TMR) comprised of 472 g of dehydrated alfalfa, 147 g of maize, 134 g of soybean meal, 76 g of barley, 53 g of beet pulp, 50 g of cereal straw, 42 g of molasses, 11 g of mineral vitamin corrector, 10 g of sodium bicarbonate and 5 g of sodium chloride per kg of mix. The chemical composition of the TMR was as follows (per kg): 909 g of dry matter, 116 g of ash, 154 g of crude protein, 2.3 g of ether extract, 328 g of neutral detergent fibre and 214 g of acid detergent fibre. Individual feed consumption was measured daily. The TMR was supplied once a day after milking; the amount of feed offered was adjusted daily on the basis of the previous day’s intake, allowing refusals of 20% of feed offered. The ewes were weighed (Magriña 102, Barcelona, Spain) on days 1 and 18 of the experimental period immediately after milking and before TMR supply.

AFB1 was purchased from Sigma-Aldrich (Merck, Rahway, NJ, USA). The mycotoxin was suspended in methanol and doses prepared by pipetting onto a wheat flour matrix contained in an oral gelatine capsule. The capsule was offered to the animals and ingested by them immediately after daily milking and before supplying the TMR, and was administered ensuring that each ewe received the correct dose of AFB1. The ewes of each group were orally supplemented from day 1 to 13 of the experimental period with different amounts of aflatoxin B1: no addition (control group C, only the amount naturally present in the ration and one capsule without AFB1 added), 40 μg aflatoxin B1 in one capsule per day (group L, low dose, 0.5 μg/kg body weight per day) and 80 μg aflatoxin B1 in one capsule per day (group H, high dose, 1.0 μg/kg body weight per day).

### 2.3. Milking

Ewes were machine-milked once a day (at 08:00) in a 1 × 10 low-line Casse system milking parlour (120 pulsations/min, 50:50 pulsation ratio, 36 kPa vacuum). Milk yield was recorded daily, and milk samples were taken on days 1, 2, 3, 4, 7 and 14. To assess AFM1 excretion after cessation of AFB1 intake, samples were taken on days 16 and 18. Two subsamples of milk were taken: one was immediately stored at −20 °C until used for AFM1 analysis and the other one was preserved (Bronopol, Broad Spectrum Micro-tabs II, D&F Control Systems, Inc., Norwood, MA, USA) and kept at 4 °C until analysed for chemical composition, which was performed within the following 24 h.

### 2.4. Blood Samples and Analyses

Blood sampling by jugular venepuncture took place on days 1, 7, 14 and 18 before offering the daily TMR. Blood samples were collected into Vacutainer tubes (10 mL; Becton Dickinson, Franklin Lakes, NJ, USA) containing either no anticoagulant or sodium heparin. Blood samples in sodium-heparin tubes were processed immediately using the automated haematology cell analyser Dymind DF50 Vet (Dymind, Shenchen, Guangdong, China) to determinate the following haematological parameters: haematocrit, haemoglobin, red blood cells, mean corpuscular volume, mean corpuscular haemoglobin, leukocytes, segmented leukocytes, eosinophils, lymphocytes and monocytes. Blood samples in tubes with no anticoagulant were allowed to clot for 30 min at room temperature and centrifuged at 2000× *g* for 15 min at 4 °C. The serum was stored at −20 °C until used to measure the metabolic profile (Analítica Veterinaria, Mungia, Spain), which consisted of: aspartate aminotransferase (AST), alanine aminotransferase (ALT), gamma glutamyl transferase (GGT), alkaline phosphatase (ALP), total protein, albumin, urea and creatinine. 

### 2.5. Physicochemical Analysis

Feed samples were analysed for dry matter [20], ash [21], crude protein [22], neutral and acid detergent fibre [23,24] and ether extract [25]. Milk samples were assayed for fat, protein and lactose concentration by automatic infrared spectrophotometry [26] using a MilkoScan 255 A/S N (Foss Electric A/S, Hillerrød, Denmark), while somatic cell counts (SCC) were assayed by a fluoro-opto-electronic technique using a Fossomatic 90 A/S N (Foss Electric A/S, Hillerrød, Denmark)).

### 2.6. Aflatoxin Analysis

Aflatoxin M1 in milk was analysed by method ISO 14501:2007 with some modifications. Milk samples were thawed, then 100 mL was warmed to 37 °C for ten minutes and then centrifuged at 4200 rpm for 30 min to separate the fat layer. The extract (lower phase) was filtered through Whatman No. 4 filter paper. About 50 mL of the filtrate was transferred into a syringe barrel attached to an immunoaffinity clean-up column (IAC Afla M1, VICAM, Watertown, MA, USA) and passed at 1–2 drops per second. The column was rinsed with 20 mL of milli-Q water for impurities removal. After that, 1.25 mL of acetonitrile/methanol (3:2 *v/v*) and 1.25 mL of milli-Q water were passed through the column to elute aflatoxin M1. The eluate was filtered with a 0.45 μm filter, placed in autosampler vials and analysed by UPLC with fluorescence detection.

The chromatographic system consisted of an Acquity UPLC H-Class system (Waters Corp., Milford, MA, USA) coupled to a fluorescence detector (2475 Multi λ Fluorescence Detector, Waters Corp., Milford, MA, USA) and controlled by Empower 3 software (Waters Corp., Milford, MA, USA). Separation was carried out on an Acquity UPLC HSS T3 column (150 mm × 2.1 mm × 1.8 μm, Waters Corp., Milford, MA, USA), with a mobile phase consisting of water/acetonitrile/methanol (68:24:8, *v/v/v*) acidified with formic acid up to pH 2.0, pumped at a flow rate of 0.2 mL/min in isocratic mode. The samples and the column were kept at 5 °C and 35 °C, respectively. The injection volume was 15 μL and the total running time was 10 min. The retention time of AFM1 was approximately 6.25 min. The wavelength of the detector was set at 360 nm (excitation) and 440 nm (emission). The detection limit was 0.92 ng/L of AFM1.

Aflatoxins in the TMR were analysed by method EN 17375:2006 with some modifications, as described by Bervis et al. [6]. Briefly, a 25 g sample was extracted with a solvent solution, filtered and diluted with water. The assay portion was passed through an immunoaffinity column, eluted with methanol, and then quantified by reversed-phase high-performance liquid chromatography (RP-HPLC) with photochemical derivatization (PHRED) followed by fluorescence detection (FLD).

### 2.7. Calculations and Statistical Analysis

Data of daily milk yield and composition, AFM1 concentration and carryover, feed intake and blood parameters were analysed using a mixed model with the fixed effects of diet, sampling day and their interaction, and the random effect of the animal nested within the treatment and the residual error. Fat- and protein-corrected milk [27] on day 0 (before the commencement of the trial) was used as a covariate in the analyses of milk yield and composition. Different covariance matrices were evaluated based on Schwarz’s Bayesian information model fit criteria. The linear mixed-effects model was performed using the lmerTest package [28] of the statistical software R version 3.3.3 [29].

The daily carryover of AFM1 in milk was calculated as the ratio between the AFM1 excreted in milk and the intake of AFB1 the previous day. Average carryover of AFM1 was calculated for the steady state (from day 4 to 13 of the experimental period). Clearance rate was calculated as the difference between AFM1 excretion on days 16 and 14 divided by that of day 14.

A stepwise regression analysis was performed (stats package of R) to select those independent parameters that helped to explain the variation in AFM1 excretion (dry matter intake, fat- and protein-corrected milk yield and AFB1 intake) and carryover rate (dry matter intake, fat- and protein-corrected milk yield, AFB1 intake and AFM1 excretion).

## 3. Results and Discussion

### 3.1. Feed Intake, Body Weight and Milk Yield and Composition

Table 1 shows that the ingestion of AFB1 (G) only affected the protein milk content (*p* < 0.05). The day of experiment (D) affected milk production and components (*p* < 0.001), but did not affect feed intake and udder health status (SCC). The G × D interaction was not significative in any case.

A decrease in milk production was observed as the experimental period progressed, although this occurred for all three experimental groups. Variations over time in milk production and composition are common in sheep and have already been described in experiments carried out under similar conditions to those described in the present study [30]. Ewes in the H group (i.e., receiving the highest dose of AFB1) had a higher protein percentage than control ewes from day 4 until the end of the experimental period (*p* < 0.05). Changes in protein percentage due to AFB1 intake are not a common effect in dairy animals. Indeed, neither sheep [14,16,31], goats [32] nor cattle [33] have suffered modifications in milk protein content in response to AFB1 administration. Therefore, this is an unexpected finding because impaired microbial protein synthesis and total protein balance in dairy animals have been previously reported [9,11]. In any case, this increased milk protein content might be also partially explained by the fact that animals in the H group exhibited numerically higher protein contents from the beginning of the experiment (6% in day 1, which become significant from day 4 onwards, with increments of 10, 9, 11 and 9% on days 4, 7, 14 and 18, respectively). 

According to previous reports [14,15,16,31], AFB1 intake did not affect milk production or composition. In the experiment carried out with Lacaune ewes [16], these even received doses of AFB1 much higher than in the present work, as they were given an average of 210 μg AFB1/animal/day for 4 weeks. On the other hand, the AFB1 doses tested in Sarda sheep without effects on productivity ranged from 2 mg in a single acute dose [14], through to 32 to 128 μg/animal per day for 1 week [15], and up to 7 μg/animal per day for 2 weeks [31]. 

In addition, no changes were observed in feed intake and body weight (*p* > 0.05), with mean body weight values being 79.8, 80.3 and 79.9 kg at the beginning and 82.3, 82.6 and 81.1 kg at the end of the experimental period for groups C, L and H, respectively. Previous reports [14,15,16,31] have also described no changes in these parameters in response to varying doses of AFB1 in the diet.

### 3.2. Haematological and Biochemical Parameters

AFB1 ingestion only affected the level of haemoglobin in the blood (*p* < 0.05). The day of the experiment affected most of the haematological parameters (*p* < 0.05–*p* < 0.001) and a great part of them presented significant G × D interactions (*p* < 0.001). In general, haematological and biochemical parameters except for blood urea remained within expected reference values for sheep [14,30,34,35]. The intake of AFB1 did not cause statistically significant changes (*p* > 0.05) in most of the analysed parameters throughout the experimental period (Table 2 and Table 3). Blood urea stood out for its high value in relation to what has been referenced (20–53 mg/dl, [34,35]). However, studies on the Lacaune breed [36] showed that this parameter is usually higher in lactating animals (69.1 mg/dl).

Haematological parameters were affected by the day of sampling; however, changes that could be detected throughout the experiment were observed in all groups of animals. No significant differences were observed between groups in white blood cells counts and distribution, as well red blood cells’ mean corpuscular volume and mean corpuscular haemoglobin (*p* > 0.10). The only effect that could be observed was a slight but significant increase in blood haemoglobin concentration (*p* < 0.05) and a tendency to increase haematocrit (*p* < 0.10) in group H animals at the end of the AFB1 ingestion period (day 14). This effect could be due to the cumulative effects of consecutive daily AFB1 intake, because the differences between groups disappeared after the clearance period (day 18). In any case, the values are within the normal range for sheep. The only recent study on this regard reported a reduction in this parameter in cows [37], whereas no changes have been reported in sheep [14] or goats [32]. This variability in response would seem to indicate that the effect would be dose- and species-dependent.

Regarding effects on blood biochemistry, most of the parameters were affected by the day of sampling, indicating an evolution over time of some of them throughout the course of the experiment in all groups of animals. Some authors have pointed out that high acute doses of AFB1 (2 mg, single dose) can lead to liver damage, usually manifested by alterations in ALP in sheep [11,14] or AST in cattle [38]. However, Battacone et al. [31] showed that low doses (up to 7 µg AFB1/day) did not lead to alterations in blood biochemistry. The animals in the present experiment received between 40 and 80 µg AFB1/day, giving a total cumulative dose of 600 and 1200 µg AFB1, which is much lower than the acute dose used by Battacone et al. [14]. Thus, the only remarkable differences were the tendency to increase in alanine aminotransferase (ALT) and total protein concentrations on day 14 (*p* < 0.10), which disappeared on day 18. This brief temporal increase could be attributable to the potential damage caused by the cumulative effects of AFB1 on liver metabolism that disappeared after the clearance period [10,11,37,38,39].

### 3.3. AFM1 Excretion

Both the ingestion of AFB1 (G) and the day of the experiment (D) affected the excretion of AFM1 (*p* < 0.001), and a significant G×D interaction (*p* < 0.001) was also observed (Table 4 and Table 5). No differences between groups were observed in the present study on day 1, coinciding with the start of AFB1 supplementation (*p* > 0.10). The increase in AFM1 concentration observed in the supplemented groups (L, H) compared to the control group was already significant (*p* < 0.001) on day 2 (first milk sampling after AFB1 supplementation), reaching a mean AFM1 concentration of 45.6 ng/L in group L, and 68.6 ng/L in group H, which confirms that the toxin ingested orally is easily absorbed in the gastrointestinal tract and rapidly metabolised [9], as observed in previous studies in sheep supplemented with AFB1 [14,15,16,31]. 

It should be noted that AFM1 excretion was highly variable both between animals of the same group within each day and between days for the same animal. The presence of AFM1 was detected in all milk samples from the control group, with a mean concentration of 3.33 ng/L. The presence of AFM1 at residual concentrations was expected, since AFB1 (0.06 μg/kg), as well as aflatoxins B2 and G1 (0.03 and 0.05 μg/kg, respectively) were detected in the basal rations of the experiment.

In the period of toxin supplementation, no statistically significant differences were observed within the same group between days 3 and 14, which could be considered a steady-state phase. This phase starts with an upward trend, reaching the maximum concentration on day 4 (79.3 and 71.9 ng/L in groups H and L, respectively). Once this maximum was reached, a downward trend was observed, with mean concentrations of 64.7 ng/L in group H and 57.2 ng/L in group L on day 14 (the first day after cessation of AFB1 supply). This kinetics, which has been described also in cattle [9,10,37] and goats [32], is very similar to those reported in sheep supplemented with doses of 23 μg, 64 μg and 128 μg of AFB1 [15]. Zhang et al. [40], in an in vitro assay, observed an increase AFM1 transporter protein gene expression followed by a gradual decrease with AFM1 incubation time. In addition, the decrease in milk yield during the experimental period may entail a reduction in the number of epithelial mammary cells and, therefore, a decrease in the transport of AFM1 to milk.

From days 14 to 16 (two days after AFB1 supply was stopped), the AFM1 concentration decreased rapidly to 8.9 and 8.3 ng/L for groups H and L, respectively, with the average clearance rates being 86 and 90% per day (3.8 and 5.1 ng/h). Clearance rates are highly variable between experiments; however, these values are within the wide range reported by other authors for dairy cattle [33,37,41]. On day 16, no statistically significant differences in AFM1 concentration in milk were observed between control and L and H groups. Likewise, the decrease in AFM1 concentration continued on day 18, as expected, when the mean concentrations were 3.27 ng/L (H group) and 2.36 ng/L (L group). These results agree with those obtained by Battacone et al. [31] in ewes fed with naturally contaminated feed, resulting in AFB1 intakes of 1.58, 3.22 and 7.07 μg/day. In ewes supplemented with 32, 64 and 128 μg of AFB1, Battacone et al. [14,15] reported that slightly longer periods (between 3 and 4 days) were needed for the disappearance of AFM1 in milk (restoration to the baseline situation).

The analysis of the regression of AFM1 concentration in milk versus AFB1 intake, milk yield and dry matter intake on the steady-state phase (once the plateau has been reached, days 2 to 14) allowed us to express AFM1 concentration in milk according to the following Equation (1):

AFM1 (ng/L milk) = 6.67 + 0.88 × AFB1 (μg/day)(1)

Residual standard error (RSE) = 23.57, R-square = 0.589, *p* < 0.001.

This means that the presence of AFM1 in milk, under the conditions of the present experiment, depends mainly on the amount of AFB1 ingested, and is not affected by the total dry matter intake or milk production. Furthermore, our results show that there may be a small amount of excretion of AFM1 in milk without being detected in the feed, something that has already been suggested by other authors [2].

Although the dose of AFB1 given to group H (80 μg) was twice that given to group L (40 μg), the excretion of AFM1 was only slightly higher in group H, and no statistically significant differences in AFM1 concentration were observed between the two groups within the same day of treatment in the plateau phase. This lack of difference is related to the carryover rate from AFB1 to AFM1, as discussed below. 

### 3.4. AFM1/AFB1 Carryover

The carryover rate of AFB1 ingested orally to AFM1 excreted in milk was calculated. In so doing, the amount of AFB1 given to each animal, the individual milk production data and the concentration of AFM1 in milk was taken into consideration (Table 6). Overall, the carryover rate was around 0.23% (AFM1/AFB1) considering the average of both, L and H groups, during the steady-state phase (days 2 to 14 of the experimental period).

Both AFB1 ingestion (G) and the day of experiment affected (*p* < 0.05 and *p* < 0.001, respectively) AFM1 carry over, but the G × D interaction was not significant (*p* > 0.10, Table 6). From day 3 until the withdrawal of the AFB1 supplementation, the transfer rate was significantly higher (*p* < 0.05) in animals given 40 μg (0.22–0.34%) compared to those receiving 80 μg (0.16–0.19%). A similar dose-response in carryover rate has been already reported in dairy cows [10]. The results obtained are also in line with those reported by Battacone et al. [15] in ewes supplemented with 32, 64 and 128 μg AFB1 (0.33%, 0.29% and 0.26%, respectively). These authors observed a decreasing trend in carryover rate, without statistical significance between groups, as the AFB1 intake increased. Further research on sheep fed with naturally contaminated feeds (AFB1 intake of 1.58, 3.22 and 7.07 μg/day), revealed higher carryover rates (2.9, 1.9 and 1.3%, respectively) than those obtained in our study. These values also differed significantly depending on the dose ingested: the higher the AFB1 intake, the lower the carryover rate [31]. Carryover rates observed for Assaf ewes are somewhat lower than those reported for Lacaune ewes (0.24–0.54% [16]), even though the latter received higher doses (up to 160 μg/day) than those in the present study. Therefore, carryover rate in sheep depends not only on the dose and the individual; the breed also seems to have a clear influence.

The observed inverse relationship between AFM1 transfer and amount of AFB1 ingested could be related to the biotransformation processes of this mycotoxin in different animal tissues. Thus, AFB1 is metabolised through complex metabolic pathways involving different enzyme systems whose activity would be modified by increasing doses of AFB1, which would eventually be metabolised and secreted by other pathways. In fact, several studies have shown that the extensive variability in the expression and catalytic activity of liver enzymes involved in the biotransformation and detoxification of AFB1 (such as cytochrome P450 and glutathione transferases) are considered to be the main cause of the differences in AFM1/AFB1 transfer found between species [9,10,11,32].

Regression analysis shows that the carryover rate (AFM1/AFB1, %) in the steady-state phase (once the plateau was reached, from days 2 to 14) can be expressed as a function of AFM1 concentration in milk, AFB1 intake and milk yield according to the following Equation (2):

AFM1/AFB1 (%) = 0.049 + 0.091 × Milk yield (L/day) + 0.003 × AFM1 (ng/L) − 0.003 × AFB1 (μg/d)(2)

RSE = 0.045, R-square = 0.887; *p* < 0.001.

Therefore, carryover rate is positively influenced by the level of milk production (probably due to increased numbers of mammary epithelial cells or AFM1 transporter proteins). Likewise, the negative influence of AFB1 may be explained by the limited AFM1 transport capacity [40].

## 4. Conclusions

In Assaf ewes that receive a daily dose of AFB1 (40 or 80 μg) for 13 days, the excretion of AFM1 in milk from starts 24 h after the first intake and depends on the dose. AFM1/AFB1 carryover is higher in animals supplemented with 40 μg AFB1 (0.22–0.34%) than in those receiving 80 μg AFB1 (0.16–0.19%). Likewise, carryover rate is positively influenced by milk yield. Once AFB1 intake has ceased, AFM1 decreases sharply, with a clearance rate of nearly 90% in the first 24 h. At the levels tested in the present study, AFB1 intake does not impair milk yield; however, it might affect animals’ health since changes in haemoglobin, ALT and total proteins were observed after 13 days AFB1 supplementation.

## Figures and Tables

**Table 1:** Mean values of feed intake, milk yield and composition for animals receiving no AFB1 or 40 and 80 µg AFB1/day (groups C, L and H, respectively) throughout the experimental period.

	G	Days					s.e.d.			*p*-Value		
		D 1 *	D 4 *	D 7 *	D 14	D 18	G	D	G × D	G	D	G × D
Feed intake (kg/animal and day)	C	3.22	3.21	3.27	3.20	3.18	0.147	0.117	0.166	0.110	0.351	0.341
	L	3.13	3.04	2.92	3.15	3.01						
	H	2.92	3.03	3.04	3.14	2.77						
Milk yield (kg/animal and day)	C	2.38	2.00	1.93	1.95	1.79	0.139	0.095	0.136	0.987	<0.001	0.977
	L	2.39	1.95	2.01	1.90	1.84						
	H	2.38	1.85	2.02	1.89	1.88						
Fat (%)	C	5.80	5.94	5.50	5.66	5.75	0.351	0.208	0.294	0.792	<0.001	0.612
	L	5.90	5.70	5.84	5.48	5.67						
	H	5.89	5.82	5.78	5.70	5.80						
Protein (%)	C	4.90	5.03 ^a^	5.07 ^a^	4.88 ^a^	4.92 ^a^	0.156	0.066	0.093	0.010	<0.001	0.565
	L	4.93	5.20 ^ab^	5.19 ^ab^	5.11 ^ab^	5.08 ^ab^						
	H	5.20	5.54 ^b^	5.53 ^b^	5.40 ^b^	5.37 ^b^						
Lactose (%)	C	4.81	4.83	4.78	4.73	4.79	0.065	0.030	0.043	0.357	<0.001	0.528
	L	4.83	4.88	4.80	4.78	4.85						
	H	4.75	4.82	4.71	4.73	4.74						
Total solids (%)	C	16.50	16.60	16.20	16.20	16.50	0.431	0.228	0.322	0.228	<0.001	0.692
	L	16.70	16.60	16.70	16.30	16.60						
	H	16.90	17.00	16.90	16.80	17.00						
Somatic cell counts (log cells/mL)	C	4.88	4.79	4.78	4.80	4.76	0.160	0.062	0.089	0.724	0.169	0.689
	L	4.85	4.81	4.91	4.87	4.88						
	H	4.99	4.92	4.94	4.94	4.85						
Total solids (g/animal per day)	C	394	331	313	316	294	20.4	15.2	21.8	0.655	<0.001	0.846
	L	398	324	335	309	304						
	H	402	314	338	315	320						
Fat and protein corrected milk (kg/animal per day)	C	2.16	1.85	1.74	1.76	1.62	0.111	0.083	0.119	0.738	<0.001	0.687
	L	2.19	1.79	1.86	1.69	1.66						
	H	2.21	1.72	1.87	1.73	1.75						

s.e.d. = standard error of the difference; G = Group; D = Day; * = Animals in groups L and H received 40 and 80 µg AFB1/day, respectively, from D 1 to D 13. ^a,b^ Different letters within the same day indicate significant differences (*p* < 0.05) between groups.

**Table 2:** Mean values of haematological parameters for animals receiving no AFB1 or 40 and 80 µg AFB1/day (groups C, L and H, respectively) throughout the experimental period.

	G	Days				s.e.d.			*p*-Value		
		D 1 *	D 7 *	D 14	D 18	G	D	G × D	G	D	G × D
Hematocrit (%)	C	28.7	28.6	29.2	29.3	1.21	0.64	0.90	0.083	0.021	0.030
	L	29.4	29.1	28.7	29.8						
	H	30.8	30.0	32.7	31.7						
Haemoglobin (g/dL)	C	10.0	10.0	10.1 ^a^	10.2	0.36	0.21	0.29	0.032	0.231	0.001
	L	10.4	10.3	10.0 ^a^	10.4						
	H	10.8	10.6	11.5 ^b^	10.7						
Red blood cells (10^6^ cells/µL)	C	8.99	9.01	9.17	9.31	0.409	0.191	0.270	0.289	0.027	0.004
	L	8.92	8.92	8.78	9.13						
	H	9.39	9.22	10.07	9.42						
Mean corpuscular volume (fl)	C	32.2	32.0	32.0	31.7	1.24	0.20	0.28	0.704	0.012	<0.001
	L	33.0	32.7	32.7	32.7						
	H	32.9	32.6	32.6	33.7						
Mean corpuscular haemoglobin (pg)	C	11.2	11.1	11.0	11.0	0.34	0.06	0.08	0.386	<0.001	0.533
	L	11.6	11.5	11.4	11.4						
	H	11.5	11.5	11.5	11.4						
Mean corpuscular haemoglobin (g/dL)	C	34.8	35.0	34.7	35.0	0.88	0.23	0.33	0.957	<0.001	<0.001
	L	35.3	35.3	34.9	34.8						
	H	35.1	35.4	35.1	33.8						
Leukocytes (10^3^ cells /µL)	C	7.69	7.63	8.20	7.79	0.957	0.398	0.563	0.993	0.007	0.723
	L	7.36	8.06	8.21	8.11						
	H	7.33	8.04	8.46	7.77						
Segmented leukocytes (%)	C	30.4	36.9	28.0	31.2	4.13	3.34	4.72	0.832	<0.001	<0.001
	L	27.4	36.1	23.4	32.6						
	H	25.2	34.4	38.2	23.8						
Eosinophils (%)	C	2.20	5.70	1.00	3.50	1.132	1.030	1.456	0.576	<0.001	0.281
	L	2.60	7.30	1.20	1.60						
	H	2.60	5.60	0.60	1.20						
Lymphocytes (%)	C	58.2	49.7	63.2	56.2	3.90	3.21	4.54	0.704	<0.001	0.001
Lymphocytes (%) Monocytes (%)	L	60.6	49.1	66.2	58.0						
	H	62.4	52.6	54.6	66.6						
	C	9.20	7.70	7.80	9.40	1.442	1.342	1.897	0.813	0.067	0.568
Monocytes (%) Segmented leukocytes (10^3^ cells /µL)	L	9.40	7.50	9.20	7.80						
	H	9.80	7.30	6.60	8.40						
	C	2.12	2.83	2.32	2.47	0.475	0.321	0.453	0.994	<0.001	0.001
Segmented leukocytes (10^3^ cells /µL) Eosinophils (10^3^ cells /µL)	L	2.00	2.98	1.93	2.67						
	H	1.87	2.77	3.19	1.77						
	C	0.171	0.447	0.067	0.236	0.0985	0.0922	0.1304	0.550	<0.001	0.554
Eosinophils (10^3^ cells /µL) Lymphocytes (10^3^ cells /µL)	L	0.208	0.607	0.085	0.129						
	H	0.182	0.447	0.051	0.096						
	C	4.41	3.76	5.17	4.31	0.606	0.317	0.449	0.888	<0.001	0.029
Lymphocytes (10^3^ cells /µL) Monocytes (10^3^ cells /µL)	L	4.46	3.87	5.44	4.68						
	H	4.53	4.23	4.64	5.24						
	C	0.670	0.595	0.644	0.749	0.1404	0.1095	0.1548	0.961	0.351	0.627
Monocytes (10^3^ cells /µL)	L	0.705	0.597	0.750	0.614						
	H	0.736	0.588	0.569	0.668						

s.e.d. = standard error of the difference; G = Group; D = Day; * = Animals in groups L and H received 40 and 80 µg AFB1/day, respectively, from D 1 to D 13. ^a,b^ Different letters within the same day indicate significant differences (*p* < 0.05) between groups.

**Table 3:** Mean values of serum biochemical parameters for animals receiving no AFB1 or 40 and 80 µg AFB1/day (groups C, L and H, respectively) throughout the experimental period.

		Days				s.e.d.			*p*-Value		
	G	D 1 *	D 7 *	D 14	D 18	G	D	G × D	G	D	G × D
Aspartate aminotransferase (AST, IU/L)	C	116	140	145	139	21.3	8.7	12.3	0.964	<0.001	0.958
	L	116	131	135	136						
	H	114	131	140	140						
Alanine aminotransferase (ALT, IU/L)	C	28.7	28.6	29.2	29.3	1.213	0.639	0.903	0.083	0.021	0.030
	L	29.4	29.1	28.7	29.8						
	H	30.8	29.9	32.7	31.7						
Gamma glutamyl transferase (GGT, IU/L)	C	48.1	69.2	65.9	59.7	8.13	5.06	7.15	0.573	0.004	0.224
	L	50.9	56.8	56.3	54.9						
	H	51.2	54.7	54.7	55.8						
Alkaline phosphatase (ALP, IU/L)	C	185	245	229	200	51.6	15.0	21.2	0.988	0.020	0.010
	L	230	227	215	218						
	H	235	233	206	206						
Total protein (g/dl)	C	6.68	6.29	6.76	7.01	0.217	0.143	0.202	0.096	<0.001	0.061
	L	6.59	6.14	6.87	6.91						
	H	6.93	6.47	7.49	7.11						
Albumin (g/dl)	C	3.39	3.28	3.49	3.52	0.163	0.092	0.130	0.575	<0.001	0.931
	L	3.46	3.31	3.60	3.67						
	H	3.47	3.42	3.68	3.72						
Urea (mg/dl)	C	66.5	59.3	60.8	59.5	3.35	2.19	3.09	0.625	<0.001	0.127
	L	66.6	61.1	61.9	64.8						
	H	71.5	62.2	62.6	59.9						
Creatinine (mg/dl)	C	0.780	0.740	0.750	0.780	0.0468	0.0351	0.0497	0.999	0.132	0.339
	L	0.740	0.760	0.770	0.780						
	H	0.800	0.700	0.780	0.770						

s.e.d. = standard error of the difference; G = Group; D = Day; * = Animals in groups L and H received 40 and 80 µg AFB1/day, respectively, from D 1 to D 13.

**Table 4:** Mean values of AFM1 excretion in milk (ng AFM1/L milk) for animals receiving no AFB1 or 40 and 80 µg AFB1/day (groups C, L and H, respectively) throughout the experimental period.

	ng AFM1/L Milk											
	D 1 *	D 2 *	D 3 *	D 4 *	D 7 *	D 14	D 16	D 18		G	D	G × D
C	2.9	2.9 ^x^	5.7 ^x^	3.3 ^x^	3.6 ^x^	3.1 ^x^	2.7	3	s.e.d.	10.16	9.14	12.92
L	2.7 ^a^	45.6 ^b,y^	55.9 ^b,y^	71.9 ^b,y^	56.2 ^b,y^	57.2 ^b,y^	8.3 ^a^	2.4 ^a^	*p*-value	<0.001	<0.001	<0.001
H	2.9 ^a^	68.6 ^b,z^	61.1 ^b,y^	79.3 ^b,y^	71.4 ^b,y^	64.7 ^b,y^	8.9 ^a^	3.3 ^a^				

s.e.d. = standard error of the difference; G = Group; D = Day; * = Animals in groups L and H received 40 and 80 µg AFB1/day, respectively, from D 1 to D 13. ^a,b^ Different letters within the same group indicate significant differences (*p* < 0.001) between days for a given parameter. ^x,y^ Different letters within the same day indicate significant differences (*p* < 0.001) between groups for a given parameter.

**Table 5:** Mean values of AFM1 excretion in milk (ng AFM1/animal and day) for animals receiving no AFB1 or 40 and 80 µg AFB1/day (groups C, L and H, respectively) throughout the experimental period.

	ng AFM1/Animal and Day											
	D 1 *	D 2 *	D 3 *	D 4 *	D 7 *	D 14	D 16	D 18		G	D	G × D
C	10.2	9.1 ^x^	13.5 ^x^	10.0 ^x^	10.5 ^x^	9.3 ^x^	8.3	8.8	s.e.d.	21.54	18.47	26.51
L	6.4 ^a^	90.2 ^b,y^	104.8 ^b,y^	136.4 ^b,y^	106.8 ^b,y^	108.9 ^b,y^	15.4 ^a^	4.2 ^a^	*p*-value	<0.001	<0.001	<0.001
H	3.5 ^a^	151.6 ^b,z^	123.7 ^b,y^	149.2 ^b,y^	150.1 ^b,y^	124.0 ^b,y^	13.2 ^a^	3.0 ^a^				

s.e.d. = standard error of the difference; G = Group; D = Day; * = Animals in groups L and H received 40 and 80 µg AFB1/day, respectively, from D 1 to D 13. ^a,b^ Different letters within the same group indicate significant differences (*p* < 0.001) between days for a given parameter. ^x,y^ Different letters within the same day indicate significant differences (*p* < 0.001) between groups for a given parameter.

**Table 6:** Mean values of AFM1 carry over (AFM1/AFB1, %) for animals receiving 40 and 80 µg AFB1/day (groups L and H, respectively) throughout the experimental period.

	D 2 *	D 3 *	D 4 *	D 7 *	D 14	D 16		G	D	G × D
L	0.224 ^b^	0.262 ^b,y^	0.341 ^b,y^	0.267 ^b^	0.270 ^b,y^	0.039 ^a^	s.e.d.	0.0529	0.0424	0.0590
H	0.194 ^b^	0.159 ^b,x^	0.191 ^b,x^	0.192 ^b^	0.160 ^b,x^	0.021 ^a^	*p*-value	0.035	<0.001	0.193

s.e.d. = standard error of the difference; G = Group; D = Day; * = Animals in groups L and H received 40 and 80 µg AFB1/day, respectively, from D 1 to D 13. ^a,b^ Different letters within the same group indicate significant differences (*p* < 0.001) between days. ^x,y^ Different letters within the same day indicate significant differences (*p* < 0.001) between groups.

## Data Availability

The data presented in this study are available on request from the corresponding author.
